# Experiences of Self-Management Support Following a Stroke: A Meta-Review of Qualitative Systematic Reviews

**DOI:** 10.1371/journal.pone.0141803

**Published:** 2015-12-14

**Authors:** Gemma Pearce, Hilary Pinnock, Eleni Epiphaniou, Hannah L. Parke, Emily Heavey, Christopher J. Griffiths, Trish Greenhalgh, Aziz Sheikh, Stephanie J. C. Taylor

**Affiliations:** 1 Centre for Technology Enabled Health Research (CTEHR), Coventry University, Coventry, United Kingdom; 2 Centre for Population Health Sciences, The University of Edinburgh, Edinburgh, United Kingdom; 3 multidisciplinary Evidence Synthesis Hub (mEsh), Centre for Primary Care and Public Health, Blizard Institute, Barts and The London School of Medicine and Dentistry, London, United Kingdom; 4 Social Policy Research Unit, University of York, York, United Kingdom; 5 Nuffield Department of Primary Care Health Sciences, Medical Sciences division, University of Oxford, Oxford, United Kingdom; University of Glasgow, UNITED KINGDOM

## Abstract

**Background:**

Supporting self-management in stroke patients improves psychological and functional outcomes but evidence on how to achieve this is sparse. We aimed to synthesise evidence from systematic reviews of qualitative studies in an overarching meta-review to inform the delivery and development of self-management support interventions.

**Methods:**

We systematically searched eight electronic databases including MEDLINE, EMBASE and CINAHL for qualitative systematic reviews (published January 1993 to June 2012). We included studies exploring patients’, carers’ or health care professionals’ experiences relevant to self-management support following a stroke, including studies describing the lived experience of surviving a stroke. We meta-synthesised the included review findings using a meta-ethnographic framework.

**Results:**

Seven reviews, reporting 130 unique studies, were included. Themes emerging from the reviews were pertinent, consistent and showed data saturation; though explicit mention of self-management support was rare. Our meta-review highlighted the devastating impact of stroke on patients’ self-image; the varying needs for self-management support across the trajectory of recovery; the need for psychological and emotional support throughout recovery particularly when physical recovery plateaus; the considerable information needs of patients and carers which also vary across the trajectory of recovery; the importance of good patient-professional communication; the potential benefits of goal-setting and action-planning; and the need for social support which might be met by groups for stroke survivors.

**Conclusions:**

The observed data saturation suggests that, currently, no further qualitative research simply describing the lived experience of stroke is needed; we propose that it would be more useful to focus on qualitative research informing self-management support interventions and their implementation. Our findings demonstrate both the on-going importance of self-management support and the evolving priorities throughout the stages of recovery following a stroke. The challenge now is to ensure these findings inform routine practice and the development of interventions to support self-management amongst stroke survivors.

## Introduction

Although the incidence of stroke may be declining in high income countries with increased life expectancy lifetime risk remains high [1] and, worldwide, stroke remains the leading cause of disability [2]. Promotion of self-management is a core response of health systems globally to the increasing burden of chronic conditions [3–6]. There is no universally accepted definition of self-management, but most definitions adopt a broad and holistic approach that encompasses self-care directed at all the aspects of life affected by a chronic condition. For example, the US Institute of Medicine define self-management as: “the tasks that individuals must undertake to live with one or more chronic conditions. These tasks include having the confidence to deal with medical management, role management and emotional management of their conditions” [7].

Ideas around actively supporting self-management were initially developed to help people with arthritis [8] and have since been extended to a wide range of chronic conditions [9]. Supporting self-management can enable patients and their families to take control of their long-term condition from diagnosis, through living with the condition, to end-of-life care. Supporting survivorship is a key element of self-management provision in chronic conditions, such as cancer [10]. A review of outcome measures of self-management interventions in stroke by Boger, Demaina and Lattera in 2013 highlights the variety of underpinning psychological mechanisms proposed and the current lack of consensus about the “concept and operation of self-management in stroke”[11]. Promoting individuals’ self-efficacy (self-confidence in relation to a specific context) is posited by some as an intermediate step that enables self-management [12]. A review by Jones and Riazi concluded that there is evidence that increased self-efficacy has a positive effect on outcomes after stroke including quality of life, health status, depression and activities of daily living, but that evidence of effective interventions to support self-management in stroke is sparse [13]. Boger et al’s review in 2015 corroborated that the majority of evidence was based around experiences of diabetes self-management and self-management support when examining stakeholders’ views of outcomes for colorectal cancer, diabetes and stroke [14]. However, this review [14] only searched for and included studies in which the terminology ‘self-management’ was explicit. There is a need to extend the evidence base by adopting a broader search and screening criterion based on the principles (as opposed to a search and inclusion criteria limited to the term) of self-management. This has already been carried out with the quantitative literature on self-management support for stroke survivors [15] and this review now addresses the qualitative literature.

Systematic reviews of qualitative studies collate and synthesise in-depth experiences and summarise the qualitative evidence in the field [16]. As part of a larger project examining the role of self-management support across a range of long term conditions [17] our meta-review aimed to summarise the qualitative literature on self-management and the lived experience of stroke to identify how self-management might be effectively supported in stroke survivors.

## Methods

### Search Strategy

We searched eight electronic databases: MEDLINE, EMBASE, CINAHL, PsychINFO, AMED, BNI, Cochrane Database of Systematic Reviews, and the Database of Abstracts of Reviews for Effectiveness. Our search strategy included the terms: ‘self-management support’, ‘stroke’ and ‘qualitative systematic review’ and a wide variety of related terms (including examples of terms we had scoped to be included under the definition of self-management support), as well as relevant MeSH terms (S1 File). We included articles published in English from January 1993 to June 2012. In addition we hand searched five journals likely to publish qualitative syntheses of stroke self-management to ensure no articles had been missed from the database searches: BMC Systematic Review, Health Education and Behaviour, Health Education Research, Journal of Behavioural Medicine and Patient Education and Counseling for the same period. The bibliographies of the included reviews were searched and *a forward citation search* was conducted in the database ISI Proceedings (Web of Science).

### Selection Criteria

We included systematic reviews of qualitative primary studies examining the lived experience of stroke, including experiences of self-management or self-management support meeting our definition [7]. We defined self-management support as: provision that aims to empower patients to be active decision makers who deal with the emotional, social and medical management of their illness with the aim of improving their independence and quality of life. Self-management support may include the provision of disease-specific information, telehealthcare, or extensive generic programmes that aim to promote behavioural change by building the confidence of individuals to manage the biopsychosocial effects of surviving a stroke [18].

We included reviews that focussed on the views of patients excluding papers only examining views of caregivers of healthcare professionals. However, if the paper examining the views of patients also included healthcare professionals and/ or informal carers perspectives then we took these into consideration for the synthesis with the aim of informing patient’s self-management support. Reviews were excluded if stroke-specific qualitative data could not be extracted independently of quantitative study data or if they failed to clearly identify (ie cite) the primary studies actually included in their review. Working independently, two reviewers (GP, HLP or EE) scrutinised all the full text papers retrieved to identify those for inclusion in the meta-review. A random 10% screening check was completed for quality assurance (ST or HP) with a 96% agreement rate of the titles and abstracts and 81% for full text screening. All disagreements were discussed as a group and final decisions about screening agreed upon together.

### Data Extraction, Quality Assessment and Analysis

In the absence of any published standards for systematically appraising the quality of qualitative systematic syntheses we adapted the R-AMSTAR (developed for assessing the quality of quantitative systematic reviews)[19] (S1 Table). Data extraction and quality assessment were carried out independently by two reviewers (GP and EH). The two reviewers had an 89% agreement rate for the qualitative version of the R-AMSTAR quality assessment. All disagreements across extraction and quality assessment were discussed and either resolved or arbitrated where necessary with a third reviewer (ST). Our approach was conservative and we excluded findings which were only reported in one systematic review if our appraisal suggested that review was of lower quality (R-AMSTAR score <30) (see S2 Table). More weight was therefore applied to reviews that were judged as higher in quality (R-AMSTAR score = 30+).

We employed a meta-ethnographic framework to synthesise these data [20]. Meta-ethnography is an established methodology for synthesising qualitative research [21]. We examined the patterns arising within these data using reciprocal translation (essentially, translating the themes arising from one study into another study) in order to organise the concepts taking place (see S3 Table and findings section). This was followed by a lines-of-argument synthesis, which is a technique used to interpret and infer at a whole level, such as organisational or cultural level, based on selective studies of different parts (e.g., studies of different groups or at different stages in a disease trajectory). In this meta-review, the lines-of-argument synthesis aimed to translate the findings into a broader understanding of their meaning in a health care commissioning context.

**Fig 1:**
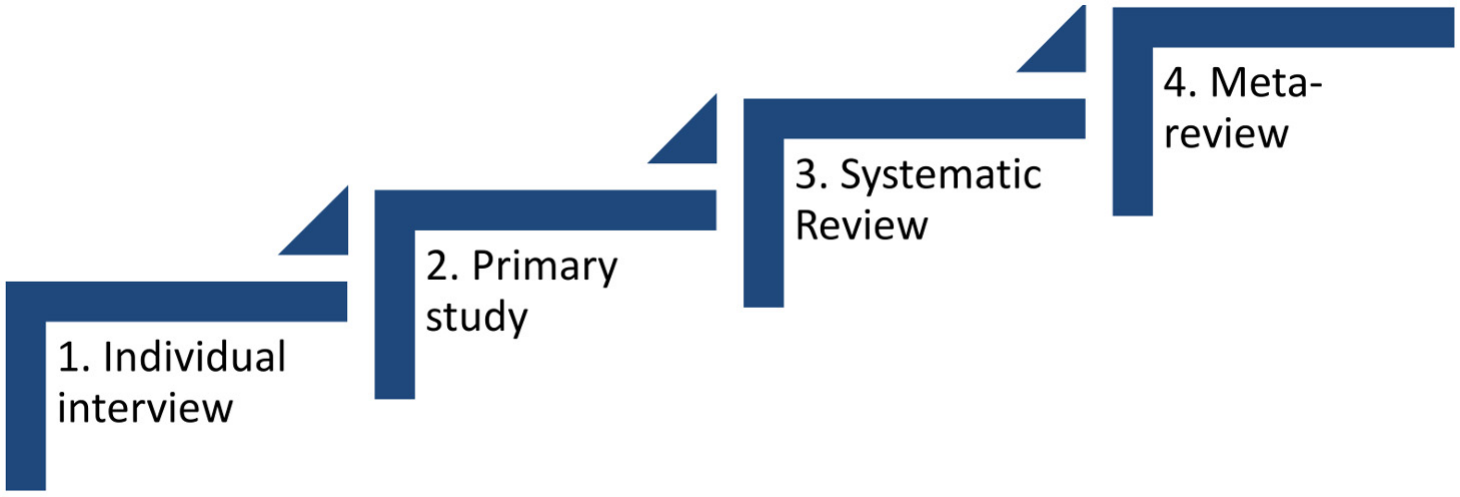
The four levels of collected data of which the meta-review is the fourth.

There are four levels of interpretation involved when conducting a meta-review of qualitative reviews (Fig 1). The first level is the participant’s interpretation of their own experiences when discussing them during the interview in the original primary research project; the second level is the researcher’s reflections and report in the primary study; the third level involves the synthesis of all the findings from the primary studies included in a systematic review; and the last is the meta-review level. In this meta-review, we focused on the second (as reported in the systematic reviews) and third levels (the findings sections of the systematic reviews) to derive the fourth level (i.e. we did not return to the individual reports of the original primary studies).

### Updating of searches prior to publication

Prior to publication, we updated our searches (May 2015) by undertaking forward citation of the original included studies. We have previously demonstrated that, in complex topic areas spanning health and social sciences, emergent approaches (e.g. citation tracking of, and pursuing selected references from, seminal papers) may be more efficient and effective than formal database searches using exhaustive search strings [22]. Accordingly, we entered the included papers from our original search into Google Scholar and manually searched the citation list for subsequent papers that met our original inclusion criteria. We considered it was very unlikely that a subsequent qualitative synthesis would be published without citing at least one of the previously published reviews. We undertook duplicate data extraction (GP and either ST or HP). Any additional themes or elaborations were integrated with the original analysis; if a major new concept had emerged we would have repeated the lines-of-argument synthesis.

## Results

**Fig 2:**
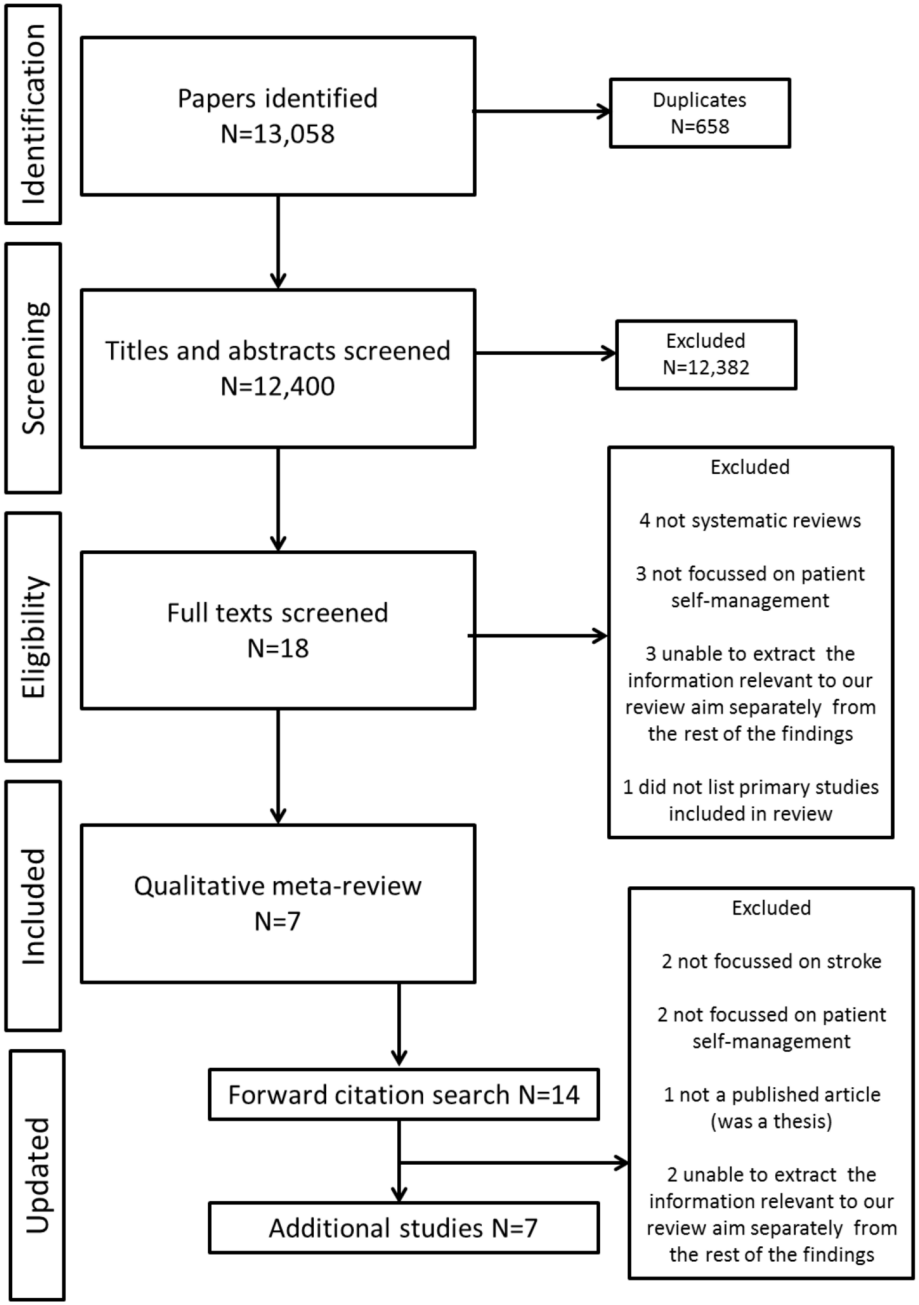
PRISMA flow chart for the meta-review and updated review results.

Our searches identified 12,400 citations, after removal of duplicates (Fig 2 for PRISMA flow diagram). Following the title and abstract screening, 18 full texts were retrieved and scrutinised and seven were included in the final meta-review [23–29]. The updating searches identified an additional 14 papers from which we selected seven as meeting the selection criteria [30–36].

### Summary of Included Studies

Of the seven reviews (published 2003–2012), six only included qualitative studies and one included a range of study designs, but reported qualitative study results separately. Some of the primary qualitative studies were included in more than one review (see S2 File for a table showing primary study overlap), there were 130 unique qualitative studies (published 1974–2008) included overall. The seven additional reviews contributed data from a further 102 unique studies.

The aims and key findings of the original review are shown in Table 1; the insights from the additional reviews are in Table 2. Four reviews in the original meta-review focussed specifically on the experiences of stroke survivors, [23, 27–29] while three discussed experiences of both stroke survivors and others who might influence their self-management [24–26]. A high level of data saturation emerged around the arising themes, suggesting similar experiences were being discussed in the majority of qualitative studies. In keeping with this observation, despite almost doubling the number of primary data sources the additional reviews generally corroborated and/or elaborated on our original findings (see Table 2): any additional insights are integrated into our presentation of results. Most reviews carried out syntheses using extraction matrices, with only three in the original meta-review and five from the updated review describing their analytical approach (clustering technique, [26] meta-summary [27], thematic synthesis [34], meta-synthesis [30,35–36], meta-ethnography [32], and meta-synthesis and lines-of-argument synthesis with a meta-ethnographic approach [28]).

**Table 1:** Summary of the included systematic review’s aim and key findings.

Author, Year, No. studies included	Review aim	Populations studied	Brief summary of key findings (NB not all reviews identified themes^a^ or similar)
**Lamb 2008 27**	To appraise and synthesise the best available evidence on the psychosocial spiritual experience of elderly individuals recovering from stroke.	Patients aged 65 or over who had experienced at least one stroke.	Four main themes arising from165 study findings: 1. A sudden unexpected event—stroke survivors perceive the stroke experience as having a sudden onset, generating shock, fear and confusion. 2. Life-altering event—individuals perceive their stroke as having life-altering consequences. 3. Connectedness—the importance of connectedness (relationships to others and to HCPs, spiritual connectedness and the lack of isolation) in the process of recovery. 4. Reconstructing life—individuals describe the recovery process as reconstructing their lives following stroke. They are engaged in the recovery which involves considerable physical and psychological work.
**Lui** ^b^ **2005 2**	To identify and review studies that have examined the effectiveness of teaching problem solving skills to caregivers in stroke care to improve patient outcomes, highlight gaps in the evidence base, and recommend avenues for additional research.	Stroke patients, caregivers and specialist nurses.	No specific themes identified but the study sheds light on the problem of unrealistic goal setting and the difference in expectations between caregivers and stroke patients. It supports the view that the goal setting process is complex, and the authors suggest there is a need to develop clearer guidelines to help nurses and family caregivers to set realistic and achievable goals.
**McKevitt200495**	To identify the scope of published qualitative studies of stroke, consider their relevance to development and delivery of services for people with stroke, and make recommendations for future work.	Stroke patients, caregivers and various groups of HCPs.	No specific themes identified; principally a mapping of qualitative research in the field rather than an overarching synthesis. Findings were discussed in four main categories: 1. Acute stroke; 2. Rehabilitation therapies; 3. Life after the acute event; 4. Community services Concludes that these studies’ contribution includes an emphasis on recording the “human” experience of stroke and the identification of needs perceived by patients and their families, differences in priorities between patients and HCPs, and barriers to best-quality care. Suggests significant problems remain in ensuring the delivery of best-quality stroke care.
**Murray2003 23**	To identify the most frequently encountered longer-term problems experienced by stroke patients and their informal carers. To provide a platform for the development of a patient-centred, primary care-based stroke service.	Stroke patients and their caregivers after discharge home following stroke.	The review identified 203 problem areas, which were categorised into five themes ^a^: 1. Hospital experience; 2. Transfer of care; 3. Communication; 4. Services; 5. Social and emotional consequences. The largest theme was the social and emotional consequences of stroke, representing 39% of all problem areas, including problems relating to mood, social changes, and attitudes to recovery, relationships and changes in self-perception. Service deficiencies, encompassing both health and social care, were the second largest theme, accounting for 29% of the problem areas.
**Peoples 201112**	To obtain the best available knowledge on stroke survivors’ experiences of rehabilitation.	Stroke patients	One overarching theme, “Power and Empowerment” with six sub-themes were identified: 1. Coping with a new situation; successful coping with the new situations (eg hospitalisation) was an important step towards the empowerment of the patient; 2. Informational needs; there was a need for sufficient, individualised need for information throughout rehabilitation; 3. Physical and non-physical needs; there was an over-emphasis on physical needs and a failure to address non-physical needs; 4. Being personally valued and treated with respect was extremely important to patients; 5. Collaboration with health care professionals; here participants’ experiences were diverse and ambivalent; 6. Assuming responsibility and seizing control was important to patients and was achieved through awareness of their situation and being involved in independent activities.
**Reed201218**	To identify the key factors to account for in planning and developing rehabilitation and community services for stroke based on users’ perspectives.	Stroke patients, caregivers and HCPs.	The main interconnected themes, or third-order constructs, relate to how the impact of stroke is influenced by: 1. the person, (stroke is a highly personalise experience, patient compare their situation with their pre-stroke selves); 2. Close social relationships; 3. The social environment (divided into the home and outside the home); and 4. The interactions between all three. The key factors to be considered in supporting stroke survivors and helping them maintain an active and positive presence in their unique social world are to: 1. identify personally relevant goals of stroke survivors and their carers, to enable personal control and independence; 2. provide practical adaptations and source appropriate levels of support to enable stroke survivors to remain in their own homes; 3. provide guidance on how to overcome the physical, economic, and psychological barriers in stroke survivors’ external worlds; and 4. enhance internal confidence by supporting positive social interaction.
**Salter20089**	To examine the contribution of the published qualitative literature to our understanding of the experience of living with stroke.	Stroke patients	Five inter-related themes were identified as follows: 1. Change, Transition and Transformation, stroke was characterised as a sudden overwhelming catastrophe which changed life irrevocably; 2. Loss, included loss of identity and loss of the pre-stroke self; 3. Uncertainty; 4. Social Isolation, the importance of relationships;5. Adaptation and Reconciliation; ideas of resilience and adaptation.

HCP = health care professional,

^a^ for consistency “domains”, “categories” and “third order constructs” are considered to be synonymous with “themes” in this table,

^b^ a mixed review with quantitative and qualitative studies, only qualitative studies reported here.

**Table 2:** Outline of the aims and additional themes from the systematic reviews added during the updating search.

Author, Year, No. studies included	Review aim	Populations studied	Existing themes^a^ corroborated by this paper	Additional themes^a^ and insights from the new paper
**Eilertsen 2012 12 studies**	To interpret and synthesise stroke survivors’ experiences of post-stroke fatigue.	Stroke survivors.	Despite the specific focus, this review corroborates the stroke survivors’ journey already described, including themes related to: Adjusting to the on-going symptoms post-stroke (and specifically fatigue), and the challenges The informational needs on coping with stroke symptoms (and specifically fatigue), and the perceived sequel and social stigma (specifically the misinterpretation of fatigue as laziness) The experiences of services, (and specifically the lack of appreciation of the limitations to rehabilitation imposed by fatigue)	This review specifically highlights the symptom of fatigue: Findings divided into two categories ‘primary / core characteristics of fatigue less influenced by context’ and those ‘additional characteristics being more responsive to the context’Lack of energy to perform activities Abnormal need for sleep Tiring easily and abnormal need for naps and rest Unpredictable feeling of fatigue Increased sensitivity to stress Fatigue was described as being invisible–‘a hidden dysfunction’ which was a hindrance in the workplace and socially.
**Gallacher 201369 studies**	To examine the qualitative literature on treatment burden in stroke from the patient perspective	Stroke survivors	This review corroborates a number of themes already found: Coping, retaining and adapting, and the need to accept the new self The evolution as physical recovery plateaus, but social goals, coping with guilt about dependency and stigmatisation become increasingly important, and challenge reintegration into society The barrier imposed by poor communication and lack of information made worse in the absence of trust in healthcare professionals	The additional contribution of this review is to consider the treatment burden of stroke (essentially the self-management strategies patients must enact in response to the demands of health care professionals and health care systems). They identified four main areas of treatment burden: “making sense of stroke management and planning care”; interacting with others (health care professionals and the community) for support (both emotional and practical); enacting management strategies (both hospital and in the community); and reflecting on their management which includes taking decisions about their care and appraising treatments. Additional specific themes are: Younger and less disabled survivors feel uncomfortable attending groups with older or more disabled stroke survivors. Those referred to a care home after hospital describes the negative experience of on-going paternalistic care. This review enlarges on the practical experience of reintegration into society: waiting for more appropriate housing and technologies to protect against falls. Lack of support to return to driving.
**Hole 2014 13** studies	To create a model of how patients ‘experiences of rehabilitation after stroke influence their outcome	Stroke survivors	Corroborates the theme of evolution over time and the need to make the transition: from the previous self and existing identity, through a complex process involving coping with and adjusting to disabilities, accompanied by a range of emotions including uncertainty, realisation, expectations, aspirations, vulnerability, isolation and hope, being supported (by peers and professionals) to rebuild and structure their social world, with the need to achieve successes/mastery to promote self-esteem and confidence to achieve integration into their social world with an ‘evolved’ identity	This review elaborates on the reintegration phase emphasising the need for professional support and positive encouragement to promote success and self-belief as patients ‘alter, adapt and evolve’ their identity during the work of reintegrating themselves into society.
**Sarre 201340 studies**	To synthesize qualitative studies on adjusting after stroke, from stroke survivors’ and carers’ perspectives, and to outline their potential contribution to an understanding of resilience	Stroke survivors and carers	This review corroborates the themes related to the impact of stroke (sudden onset, with effects not only on physical disability, but also sense of self and relationships) and the process of adjusting to the effects which unfolds over time. There is a need for recovery, and then adjustment and/or resilience as stroke survivors seek to achieve (a new) ‘normality’. It also identified the many factors which influence the success of this process, including social, healthcare and work-related support.	This review elaborates on the theme of reintegration into society and explicitly highlights that the ability to return to work depends on the willingness of the employer to adapt the workplace as well as the attitudes of other employees. Disability discrimination legislation was important.
**Satnik 2013 33 studies**	To synthesize patients’ views on the impact of stroke on their roles and self	Stroke survivors	This review corroborates the themes relating to the challenges of regaining or developing a new self which they trust and with which they feel comfortable. There is a need to identify new roles that were valued, enabling them to contribute to family/work/society despite having to adapt. For some this involved adapting work arrangements (e.g. by reducing stress, taking rests, reducing hours), others changed their social position, such as decided to spend more time with their grandchildren	This review elaborates on themes related to reintegration including the need for stroke survivors to move from passively being ‘cared for’ post-stroke to actively managing their own evolution to social reintegration. Paternalism from healthcare professionals and overprotection from family were cited as barriers to autonomy and actively taking control.
**Walsh 2014 18 studies (2 were mixed methods with qualitative findings extracted only)**	To examine the barriers and facilitators of community reintegration in the first year after stroke from the perspective of people with stroke	Stroke survivors	This review corroborates the themes related to: Barriers to social reintegration, especially (sometimes fluctuating) physical disabilities, emotional challenges (including loss of confidence and fear of recurrence especially if previous lifestyle was perceived to be reason for stroke) and issues of stigma and acceptance by others. Positive psychological characteristics (e.g. hope, resilience, perseverance, risk taking) increased success in recovery. Healthcare professional/patient relationship needed to maintain momentum after early physical recovery with need for better communication about return to work, driving, and fatigue management. Some patients experience a sense of abandonment by healthcare professionals.	This review elaborates on the theme of community reintegration: Stroke survivors need to find ways to help others in order to reduce their sense of being a burden Returning to driving is an important facilitator; there were often difficulties with public transport. Rehabilitation in hospital/institutional settings was not always perceived as being relevant to real-life when back at home. It is important to strike a balance between capacity, identity and personal expectations.
**Williams 20136 studies**	To explore the experience of engaging in occupation following a stroke for older people in the community?	Stroke survivors	This review corroborates themes related to: Emotional impact of stroke and the need to seek a new ‘self’, the loss of social contact and the need to develop new relationships (e.g. stroke/community support group)	Additional elaboration includes: Inaccessible environments and a specific fear of falling The wish to retain meaningful occupations.

HCP = health care professional,

^a^ for consistency “domains”, “categories” and “third order constructs” are considered to be synonymous with “themes” in this table,

^b^ a mixed review with quantitative and qualitative studies, only qualitative studies reported here.

### Quality

In the original meta-review, we assessed four papers as higher quality, [23, 26, 28, 29] with the remaining three having lower scores [24–27] (see S2 Table).

### Synthesis

The qualitative syntheses included in the meta-review provided an overview of a stroke survivor’s journey: from the acute stroke, to rehabilitation, to looking towards the future. Three central themes pertinent to self-management support were identified: impact of stroke; needs as a result of stroke; and the experience and impact of services (see S3 Table for exemplar quotations from the systematic reviews). These are presented under the following headings related to our aims:

1. The qualitative literature on self-management in stroke
2. The lived experience of stroke: a) impact of stroke and b) needs as a result of stroke
3. How self-management might be effectively supported in stroke survivors: experience and impact of services.

These are presented from the original meta-review, with findings from the updated reviews added when these provide further information.

### Qualitative literature on self-management in stroke

None of the included reviews explicitly examined self-management support interventions in stroke survivors. Instead they examined patients’ experiences of being a stroke survivor, either more generally or with a specific focus on psychosocial experiences in the elderly population [23, 25, 27, 29]. They also examined people’s views of services offered to stroke survivors, investigating challenges faced and potential solutions found [24–28].

Despite the lack of self-management support terminology, many of the themes described below were pertinent across all of the included reviews, especially the over-arching focus on enabling the stroke survivor to achieve independence; not only physically as functional recovery is optimised, but also psychologically as the emotional aspects of living with stroke are addressed and socially as the individual is supported to achieve the longer-term goal of regaining a meaningful role within society.[30–36]

### The lived experience of stroke

#### Impact of stroke

This theme relates to the disruption of having a stroke and the impact this has on a person’s life. Having a stroke was a sudden shocking event that the stroke survivors perceived had disrupted their anticipated life trajectory [23, 29]. The event was associated with feelings of loss, grief, frustration, embarrassment, shame due to physical and social changes [23, 25–29] and helplessness [23, 29]. Fatigue was an ‘invisible’, and often unpredictable, problem that was poorly understood by family and healthcare professionals, but which challenged social reintegration.[30] People who had experienced a stroke viewed their bodies as unreliable and their futures as uncertain. They compared themselves to their pre-stroke selves, leading to further feelings of frustration and anxiety [23, 25–29]. Additionally, in Reed’s [28] review, stroke survivors reported feeling safe in their home environment, but unsafe and vulnerable outside the home, intensifying separation from the outside world. Stroke survivors reported the stroke as resulting in a new self, with recovery involving acceptance of the changes in their lives and self-image, reassessment of role and reconstruction of their identity [23, 26, 28, 29].

#### Needs as a result of stroke

Inherent in this theme stroke survivors reported physical, informational, psychological, and social needs. Initial recovery was predominantly focussed on medical management and physical rehabilitation. Basic aspects of life which patients had previously taken for granted, such as getting out of bed, walking or talking, now involved a conscious effort that permeated and affected other aspects of their lives [23, 27–29]. Early discharge was welcomed as patients felt more in control at home, and able to assess their needs and carry out their own routines, as opposed to passively complying with healthcare schedules while inpatients [25, 27, 28]. The reviews recommended that support should be personalised to help the individual carry out daily tasks, to increase feelings of confidence, independence and hope, and in turn further motivate the survivor to set and achieve new, realistic challenges [23–29].

Stroke survivors reported a lack of understanding about having a stroke and life afterwards, and requested further guidance and information on what they were experiencing and what to expect at different stages of stroke survival and how best to self-manage [23, 25–29]. In some cases, stroke survivors reported feeling afraid of having another stroke and consequently curtailed their activities to an extent that could derail recovery [23]. The reviews suggest that health care professionals should be specifically trained to empower stroke survivors by exploring their understanding of stroke, identifying barriers to adjusting and self-managing, and providing patient-specific information at all stages of recovery [23, 25–29].

Stroke survivors described rehabilitation as focussed on functional rehabilitation and not supporting their psychological and emotional needs [23, 25–29]. The reviews highlighted that goal setting and problem solving should be patient-centred, educating the stroke survivor on how to set their own realistic goals and take steps to achieve these. This, in turn, provided the stroke survivor with the self-efficacy to apply these skills when faced with new challenges [23–29]. Stroke survivors often found it frustrating trying to relearn something at which they were once highly skilled, whereas developing new skills could be perceived as a productive and enjoyable challenge [23]. Returning to driving was a practical facilitator which was seen as the key to re-establishing social connections.[31, 35]

Many stroke survivors felt isolated as they faced many barriers in their struggle to reintegrate into a meaningful role in the community [35, 36]. They missed their previous social lives and felt that those around them did not understand what they were going through [23, 25–29]. They often felt the need to ‘manage’ the impressions they gave to other people and tried to match the public perception of what a ‘normal’ person should be like, in order to avoid negative perceptions and stigma [29]. This, in turn, could be emotionally draining and could lead to further feelings of isolation. Social support groups with other stroke survivors and people who were understanding and supportive were reported as valuable for exchanging experiences and coping strategies. These encouraged comparison with other people in similar situations, rather than comparison with their pre-stroke selves or those who had not experienced a stroke [23, 25–29]. This reduced feelings of isolation, concerns of impression management and social vulnerability, and increased feelings of self-efficacy in social situations.

Some stroke survivors reported that carers could be overprotective and reduced their autonomy [26,34]—suggesting that self-management support should encompass family, friends and the stroke survivor’s social network. A positive outcome of stroke self-management should be to enable the stroke survivor to feel comfortable within their social world regardless of the residual effects of the stroke [28]. The ability to return to work depended on the willingness/ability of the employer to adapt the workplace as well as the attitudes of the other employees [33]. Reviews suggested that rebuilding these social links and survivors’ psychological (and sometimes spiritual) strength could help increase feelings of belonging, connectedness, confidence, independence and hope, enabling the stroke survivor to refocus their life and look towards their future [23, 25–29,32].

How self-management might be effectively supported in stroke survivors: experience and impact of services Within this theme, stroke survivors discussed four aspects relating to their experiences of services and the impact this had on their recovery: the recovery plateau; communication; respect; and proactivity (defined as creating or controlling a situation rather than just responding to it). The recovery plateau is a result of treatment focussing on the physical and medical aspects of recovery. Following a stroke, survivors received rehabilitative support for a period during which they observed physical improvements. Typically these improvements then plateaued and this was accompanied by the withdrawal of active rehabilitation treatment [25, 29]. This plateau in physical recovery often occurred simultaneously with the self-realisation that patients were not going to reach what they perceived to be a full recovery (i.e. returning to their pre-stroke self) [25, 29]. At this pivotal point, patients described a lack of psychological and social support to enable them to accept these changes to their self-perception and identity [23, 26, 28, 29]. The reviews found that relationships with healthcare professionals should be collaborative and educational, with the professional listening to the patients’ and carers’ needs, giving expert advice, providing information and answering questions [23–28].

Stroke rehabilitation is ‘very demanding’ for patients with significant burdens imposed by the treatment schedules [31]. One of the most commonly reported issues was a mismatch of goals, or different perceptions of the same goals, between the stroke survivor and the healthcare professional. These were often caused by differing views of the concept of recovery, how it could be attained and how progress towards it might be measured [23–29]. Stroke survivors reported receiving mixed messages from different professionals about what to do and what not to do leading to confusion and feelings of guilt if they were not doing something or improving at a certain rate [27]. However, they reported that being taught skills that enabled more proactive engagement, such as goal setting, resulted in autonomy, control and empowerment [23–29].

Stroke survivors tended to view professionals as experts and expected them to take on a controlling role. In the early stages of recovery, patients sometimes reported feeling relieved from the burden of decision making [23,27]. However, care routines were thought to constrain autonomy and stroke survivors reported feeling as though they were waiting passively for treatment [27]. Even those who felt that their self-management skills were improving reported that they missed professional support when it was reduced [23]. Generally, stroke survivors discussed the need to feel valued, and reported that this was hindered whenever carers or professionals treated them with a lack of respect and dignity [23, 25, 27, 29]. This resulted in feelings of subordination and a lack of control over their own lives. Stroke survivors reported feeling invisible and isolated to those around them yet, paradoxically, they also felt that they were highly visible as a burden to those who were caring for their vulnerable selves [23, 25, 27, 29]. They reported that feeling respected and valued helped build communication between the professional and patient. Feeling emotionally and socially supported (rather than isolated and a burden) could enable the survivor to feel confident in their recovery and encouraged them to take a proactive approach to self-management. It was reported as important that all those involved with the stroke survivor’s management (e.g., informal carers) are involved in this process and come to a consensus about realistic targets in the short and long term [23, 25–29].

## Discussion

This meta-review brings together a vast amount of qualitative research (including data from 232 unique studies) on the experience of stroke and relating to the ways patients, carers and health professionals perceive self-management might be supported. Themes emerging from the reviews were pertinent, consistent and showed data saturation; though explicit mention of self-management support was rare. Our meta-review highlighted the devastating impact of stroke on patients’ self-image; the fact that informational, physical, psychological and social support needs evolve as the person moves through the different stages of recovery from acute stroke, to rehabilitation, to being a stroke survivor and looking towards the future; and the potential benefits of goal-setting and action-planning underpinned by good patient-professional communication potentially supported by groups for stroke survivors.

### Discussion in relation to other published literature

Our other meta-review [15], examining the quantitative evidence in this field, highlighted that the term ‘self-management’ was poorly recognised and infrequently utilised. Instead, other interventions that support self-management, but were not explicitly described as such, were found, often therapy-based rehabilitation. Although practitioners, such as occupational or other therapists, aim to address the medical/behavioural, emotional and role-related needs proposed by Corbin and Strauss, [37] current provision for those living with stroke appears to focuses more on supporting medical/behavioural needs, with less support being provided for on-going emotional and role needs. Stroke survivors still struggle when support is withdrawn once their physical recovery has plateaued, in part because they may lack confidence about recovery and are uncertain how to approach future challenges that may arise. Self-management support could focus on building self-efficacy to help stroke survivors feel more empowered [11,13]. As social modelling is a source of self-efficacy, social groups can be an important source of support for stroke survivors, especially as their physical recovery begins to plateau. Building these social relationships can help reduce concerns around the stigma they associate with having a stroke [38] and their need to manage the impression they create [39–41].

These findings corroborate with the conclusions of McCorkle et al.[10] regarding cancer survivorship, that strong collaborative relationships are needed between patients and professionals to create mutually agreed care plans to empower the patient to self-manage effectively. Conditions which result in biographical disruption [42–43] may cause the individual to compare their old non-disrupted self to this new self. Healthcare professionals can enable the stroke survivor to cope with the changes and adjust positively to their new identity. They can involve family and friends so the stroke survivor feels supported. The recommendation to involve social support in the adjustment to change is supported by the findings of Levack, Kayes, and Fadyl’s [44] qualitative meta-synthesis of services provided to adults with traumatic brain injury. Levack, et al. found that social and emotional support was important to those experiencing feelings of disconnect to their bodies, identities and to other people as a result of a traumatic brain injury. Additionally, other recommendations are generic, such as the need to be treated with respect and feel valued; the need to match the goals of patients, professionals and informal carers; and the perceived success of the stroke survivor when feeling proactive and empowered in recovery.

Although there was not an explicitly focussed qualitative review on the experiences of self-management support, there is much qualitative research in existence regarding the experiences of living with a stroke and current services provided for those who have had a stroke. Findings reached a high level of data saturation suggesting that there is no need for further qualitative synthesis: the focus now should be to use these insights to inform the development of interventions to support routine care for those surviving stroke.

### Limitations and strengths

We recognise that a meta-review distances the reviewers from the primary material losing depth and reflexivity at the primary level. Limited word counts for systematic reviews may also result in truncation and loss of depth in their syntheses. We took a conservative and transparent approach only reviewing what was reported by the included systematic reviews and conducting an explicit process of thematic analysis from the constituent reviews. Multiple reviewers carried out each stage of the review to address any potential areas of subjectivity, [45] discrepancies were discussed and consensus was reached. We also acknowledge the limitation that only including systematic reviews might have meant more recent primary study findings were missed from this meta-review, though we updated our searches prior to publication, which corroborated and/or extended the central themes from our original analysis. The high level of data saturation with only a modest overlap in primary studies between reviews gives us confidence in the credibility of the results. In the absence of a quality assessment tool validated for qualitative meta-syntheses, we adapted the R-AMSTAR which was designed for quantitative meta-reviews [19].

only three reviews in the original meta-review [26–28] and five in the updated review [30,32,34–36] discussed the type of methodology employed to collect and synthesise the qualitative studies. A potential reason for this may be the large range of qualitative syntheses methodologies available and an understanding of the epistemologies needed to carry out these reviews. There are papers that detail a range of both mixed methods reviews [16] and qualitative syntheses [21] in order to inform researchers. However, when carrying out a meta-review of qualitative synthesis, unless reviewers label the method used, it is difficult to assess the epistemology followed. There is also the issue of misrepresentation of methods in a lower quality review, which can be addressed by a compendium of syntheses [21]. This was beyond the scope of this meta-review but would be a beneficial tool in the future.

## Conclusions

The findings from this meta-review highlight the importance of support for self-management at all stages of recovery following a stroke. Key recommendations relating to the provision of self-management support focus on four aspects: a) recognition that the support needs of stroke survivors change over the trajectory of recovery b) the importance of collaborative relationships with healthcare professionals who offer guidance and information specific to the stage of recovery and enable patients to feel respected, valued, and in control of decisions about their lives; c) the provision of individually tailored psychological, emotional and behavioural support from the early stages after suffering a stroke to proactively help stroke survivors manage the long-term challenges of reintegration into society by increasing adaptive coping strategies and help them adjust to their new self by focusing on new or developing skills; and d) social groups with other stroke survivors and groups of people who are understanding and supportive. The challenge now is to incorporate these findings into routine practice and to develop novel interventions to effectively support self-management. We recommend a move from examining the qualitative experience of living with a stroke, to focussing on informing the mechanisms of improving, mobilising knowledge and supporting the self-management of people who have had a stroke.

## Supporting Information

S1 File The full MEDLINE search strategy. (DOCX)

S2 File Study overlap within the included reviews. (DOCX)

S1 Table Qualitative Meta-Review Quality Assessment Tool. (DOCX)

S2 Table Stroke quality assessment results for qualitative systematic reviews. (DOCX)

S3 Table Stroke themes and example quotations from the qualitative reviews. (DOC)
