# Supplementary material for: Experiences of Self-Management Support Following a Stroke: A Meta-Review of Qualitative Systematic Reviews
Source: PLoS One. 2015 Dec 14;10(12):e0141803. doi: 10.1371/journal.pone.0141803 (PMC4682853; doi:10.1371/journal.pone.0141803)
Supplement: S1 File — (DOCX) [file pone.0141803.s001.docx]

**Supporting Information S1: full MEDLINE search strategy**

1. Exp Self care/
2. Exp Communication/
3. Exp Professional Family Relations/
4. Exp Telephone/
5. Exp Professional Patient Relations/
6. Exp Health education/
7. Exp Attitude of health personnel/
8. Exp Cellular phone/
9. Exp Patient education as topic/
10. Exp Handheld computer/
11. Exp Self efficacy/
12. Exp Activities of Daily Living/
13. Exp Self help devices/
14. Exp Community health services/
15. Exp Rehabilitation/
16. (Self ADJ2 (car* or manag* or help or admistrat* or monitor* or medicat*)) or self-car* or self-manag* or self-help or self-adminisrat* or self-monitor* or self-medicat* or selfcar* or selfmanagement or selfhelp or selfadministrat* or selfmonitor* or selfmedicat* or SM.ti/ab.
17. Responsib* or Autonom*.ti/ab.
18. Manag* or copes or coping.ti/ab.
19. “Disease management”.ti/ab.
20. “expert patient”.ti/ab.
21. (professional or clinician) ADJ2 development.ti/ab.
22. Educat* or training or skill* or knowledge.ti/ab.
23. Confidence or self-efficacy.ti/ab.
24. (Access* or provi*) ADJ3 (information or records or results).ti/ab.
25. Monitor* or self-monitor* or selfmonitor*.ti/ab.
26. ((patient or individual* or person* or client*) ADJ3 (remind* or feedback)).ti/ab.
27. (Tele ADJ2 (health or medicine or care)) or tele-health or tele-medicine or tele-care or telehealth or telemedicine or telecare.ti/ab.
28. “Short message service” or SMS or “mobile phone” or “text message*”.ti/ab.
29. (home or environment* or living or assistive) ADJ2 (adaptation or modif* or equipment or technolog*).ti/ab.
30. “Care plan*”.ti/ab.
31. “Action plan*”.ti/ab.
32. Hypno* ADJ1 (self or home)ti/ab.
33. (cognitive or psychological or interpersonal or relaxation or biofeedback) ADJ3 (therap* or intervention* or program*).ti/ab
34. CBT.ti/ab.
35. Psychoeducation*.ti/ab.
36. (Peer or patient or emotional or social or psychosocial) ADJ1 (support or group) .ti/ab.
37. Financial ADJ1 control.ti/ab.
38. “personal health budget*”.ti/ab.
39. (Financial or monetary or payment* or discount or service*) ADJ5 incentiv*.ti/ab.
40. Exercise or training or rehabilitati*.ti/ab.
41. (Lifestyle or occupational) ADJ1 (intervention* or modification* or therapy) .ti/ab.
42. “Speech and language therapy”.ti/ab.
43. Or/1-42
44. Stroke/
45. Brain ischemia/
46. (Stroke or poststroke or post-stroke or cerebrovascu* or “brain vasc*” or “cerebral vasc*” or cva* or apoplexy* or sah) .ti/ab.
47. ((brain* or Cerebr* OR vascular OR cerebell* or intracran* or intracerebral* or subarachnoid) ADJ1 (accident OR isch?mi* OR infarct* or thrombo* or emboli* or occlus* or h?morrhage or h?matoma* or bleed*)).ti/ab.
48. Or/44-47
49. meta-analysis/
50. meta analysis as topic/
51. Review literature as topic/
52. MEDLINE.ti/ab.
53. (systematic review* or meta-analy* or metaanaly* or "research synthesis" or literature review) .ti/ab.
54. systematic ADJ3 literature.ti/ab.
55. data ADJ2 extract*.ti/ab.
56. ((information or data) ADJ3 synthesis).ti/ab.
57. Cochrane.ti/ab.
58. (qualitative or narrative or thematic or evidence or realist or interpret* or induct* or refutational or framework or systematic or textual) adj2 (approach or review* or synthes* or meta-summary or “meta summary” or summary).ti/ab.
59. Meta adj1 (summary or narrative or synthesis or ethnograph* or study or data or interpretation or aggregation or needs-assessment or “needs assessment”).ti/ab.
60. meta-summary or meta-narrative or meta-synthesis or meta-ethnograph* or meta-study or meta-data-analysis or meta-data-synthesis or meta-interpretation or meta-aggregation
61. “reciprocal translational analysis”.ti/ab.
62. RTA.ti/ab.
63. “lines-of-argu?ment synthesis” or “lines of argu?ment synthesis”.ti/ab.
64. “LOA synthesis”.ti/ab.
65. “grounded formal theory”.ti/ab.
66. “grounded theory synthesis”.ti/ab.
67. ecological adj2 (triangulation or sentence or synthesis).ti/ab.
68. Phenomenography.ti/ab.
69. ((mixed or multi* or cross) adj1 (method* or design* or research or strategy)) adj2 (synthesis or review).ti/ab.
70. (mixed-method* or multi-method* or mixed-design or multi-design or multiple-methods or multi-strategy or cross-design) adj2 (synthesis or review).ti/ab.
71. “research synthesis”.ti/ab.
72. Data ADJ2 extract*.ti/ab.
73. ((information or data) ADJ3 synthesis).ti/ab.
74. Bayesian adj1 (meta-analysis or “meta analysis”).ti/ab.
75. “case survey”.ti/ab.
76. “qualitative comparative analysis”.ti/ab.
77. Or/49-76
78. letter.pt
79. comment.pt
80. editorial.pt
81. Or/78-80
82. 77 not 81
83. 43 and 48 and 82

NB. Search strategy for systematic reviews of qualitative studies was combined with systematic reviews of RCTs. The latter was full text screened, synthesised and written up separately.
