# Supplementary material for: Experiences of Self-Management Support Following a Stroke: A Meta-Review of Qualitative Systematic Reviews
Source: PLoS One. 2015 Dec 14;10(12):e0141803. doi: 10.1371/journal.pone.0141803 (PMC4682853; doi:10.1371/journal.pone.0141803)
Supplement: S2 File — (DOCX) [file pone.0141803.s002.docx]

**Supporting Information Table S5**: Study overlap within the included reviews

|  | Lamb et al., 2008 | Lui, et al., 2005 | McKevitt, et al., 2004 | Murray, et al., 2003 | Peoples, et al., 2011 | Reed, et al., 2012 | Salter, et al., 2008 |
| --- | --- | --- | --- | --- | --- | --- | --- |
| Lamb et al., 2008 | 27 |  |  |  |  |  |  |
| Lui, et al., 2005 | 0 | 2 |  |  |  |  |  |
| McKevitt, et al., 2004 | 16 | 2 | 95 |  |  |  |  |
| Murray, et al., 2003 | 3 | 2 | 15 | 23 |  |  |  |
| Peoples, et al., 2011 | 3 | 0 | 4 | 0 | 12 |  |  |
| Reed, et al., 2012 | 5 | 0 | 7 | 2 | 0 | 18 |  |
| Salter, et al., 2008 | 5 | 0 | 8 | 3 | 0 | 3 | 9 |

The numbers in the shaded cells indicate the total number of studies included in each review. The numbers in the unshaded cells indicate the number of studies common to each corresponding pair of reviews.
