# Supplementary material for: Experiences of Self-Management Support Following a Stroke: A Meta-Review of Qualitative Systematic Reviews
Source: PLoS One. 2015 Dec 14;10(12):e0141803. doi: 10.1371/journal.pone.0141803 (PMC4682853; doi:10.1371/journal.pone.0141803)
Supplement: S1 Table — (DOCX) [file pone.0141803.s003.docx]

**S1 Table: Qualitative Meta-Review Quality Assessment Tool^a^**

These decisions were based upon the information provided in the published report; no further information was sought from the reviewers. It was therefore considered to be the judgement of the quality and explicitness of their reporting. Each main question has smaller sub-questions to guide the answer to the main question. Each question is positively worded so if the answer is yes, it will be considered as a positive towards the assessment of the review’s quality.

**Appropriate and detailed design provided?**

1) Does the review specify their original protocol with details of any iterative changes made explicit and the rationale for these changes explained?

2) Do they provide a statement of inclusion criteria?

3) Do they use PICO to guide their research question(s)?

**Duplicate study selection & data extraction?**

1) Did they have at least 2 independent data extractors?

2) Did they have a consensus procedure for disagreements?

3) Were disagreements resolved properly?

**Did they carry out a comprehensive literature search?**

1) Did they search at least 2 electronic sources?

2) Did they state both the years and databases that they searched?

3) Did they state key words/MESH terms and were you able to trace the filtering of articles for inclusion, for example a flow diagram?

4) Were textbooks/experts/references of included reviews consulted?

5) Did they hand-search/manually-search journals?

**Was status of publication used as an inclusion criterion?**

1) Did they state whether they searched for reports regardless of publication type?

2) Did they state if they excluded reports based on publication status, language etc?

3) Did they state whether non-English papers were translated?

4) Did they include all languages in the review (have no language restriction)?

**Was a list of included and excluded studies provided?**

1) Did they provide a table/list of included studies?

2) Did they include a table/list of excluded studies after full text screening?

3) Do they state the reason for exclusion of studies at the full text screening stage?

4) Are you able to retrace included and excluded papers through references?

**Were characteristics of included studies provided?**

1) Did they provide summarised information on participants, methods, contexts?

2) Did they provide ranges of relevant characteristics provided eg age, gender, ethnicity?

3) Did the information provided appear to be complete and accurate?

**Was scientific quality of included studies assessed and documented?**

1) Did they have an *a priori* method of assessment?

2) Did the scientific quality of included studies appear meaningful?

3) Did they include a discussion/awareness of levels of evidence?

4) Did they score the quality of included studies using a set instrument/technique?

**Was scientific quality of included studies used appropriately in formulating conclusions?**

1) Were the results of quality and credibility considered in the analysis and conclusions?

2) Were the results of quality and credibility explicitly stated in formulating recommendations?

3) Do the conclusions drive towards a clinical consensus statement/summary of evidence?

4) Does this clinical consensus statement drive towards a recommendation for practice?

**Were appropriate methods used to combine findings of studies?**

1) Did they provide a description of how the primary studies were analysed?

2) Was the type of analysis appropriate to answer the research questions/achieve the aims?

3) Do they discuss convergence within the primary study’s findings?

4) Do they discuss divergence within the primary study’s findings?

**Was conflict of interest stated?**

1) Was a statement of sources or support provided?

2) Did they state that there was no conflict of interest?

3) Did they state an awareness of conflict of interest in the inclusion of primary studies, i.e., providing a discussion on author reflexivity?

^a^ Adapted from: Kung J, Chiappelli F, Cajulis OO, Avezova R, Kossan G, Chew L et al. From Systematic Reviews to Clinical Recommendations for Evidence-Based Health Care: Validation of Revised Assessment of Multiple Systematic Reviews (R-AMSTAR) for Grading of Clinical Relevance. The Open Dentistry Journal 2010; 4: 84-91.
