# Supplementary material for: Experiences of Self-Management Support Following a Stroke: A Meta-Review of Qualitative Systematic Reviews
Source: PLoS One. 2015 Dec 14;10(12):e0141803. doi: 10.1371/journal.pone.0141803 (PMC4682853; doi:10.1371/journal.pone.0141803)
Supplement: S2 Table — (DOCX) [file pone.0141803.s004.docx]

**S2 Table: Stroke quality assessment results for qualitative systematic reviews (highest to lowest rated from left to right – scores under 30 given lower weighting)**

| **R-AMSTAR Criteria** | **Reed et al, 2012** | **Lamb et al, 2008** | **Murray et al, 2003** | **Salter et al, 2008** | **Lui et al, 2005** | **Peoples et al, 2011** | **McKevitt et al, 2004** |
| --- | --- | --- | --- | --- | --- | --- | --- |
| Was an appropriate and detailed design provided? | 4 | 4 | 4 | 4 | 4 | 4 | 4 |
| Was there duplicate study selection and data extraction? | 4 | 3 | 4 | 4 | 3 | 1 | 2 |
| Was a comprehensive literature search performed? | 3 | 2 | 4 | 4 | 3 | 4 | 4 |
| Was the status of publication (i.e. grey literature) used as an inclusion criterion? | 3 | 4 | 2 | 1 | 2 | 2 | 2 |
| Was a list of studies (included and excluded) provided? | 4 | 4 | 2 | 2 | 1 | 2 | 2 |
| Were the characteristics of the included studies provided? | 4 | 1 | 4 | 2 | 4 | 2 | 2 |
| Was the scientific quality of the included studies assessed and documented? | 4 | 4 | 1 | 4 | 1 | 1 | 1 |
| Was the scientific quality of the included studies used appropriately in formulating conclusions? | 4 | 4 | 4 | 4 | 4 | 2 | 1 |
| Were the methods used to combine the findings of the studies appropriate? | 4 | 4 | 4 | 4 | 3 | 4 | 4 |
| Was the conflict of interest stated? | 3 | 2 | 1 | 1 | 1 | 4 | 2 |
| **Total score/40** | **35** | **32** | **30** | **30** | **26** | **26** | **24** |
| **Quality Rating** | **High** | **High** | **High** | **High** | **Low** | **Low** | **Low** |
