# Supplementary material for: Experiences of Self-Management Support Following a Stroke: A Meta-Review of Qualitative Systematic Reviews
Source: PLoS One. 2015 Dec 14;10(12):e0141803. doi: 10.1371/journal.pone.0141803 (PMC4682853; doi:10.1371/journal.pone.0141803)
Supplement: S3 Table — (DOC) [file pone.0141803.s005.doc]

**S3 Table**: Stroke themes and example quotations from the qualitative reviews

| **Central themes** | **themes** | **Reference** | **Illustrative quotation** |
| --- | --- | --- | --- |
| Impact of stroke | Shock | Lamb et al., 2008 | ‘Stroke survivors perceive the stroke experience as having a sudden onset, generating shock, fear and confusion.' (p177) |
|  |  | Salter et al., 2008 | ‘Stroke was characterized as a sudden and overwhelming catastrophe, a fundamental life change and profound disruption; one which separated survivors from their everyday life and forced them into a new and foreign existence.' (p597) |
|  | Negative change | Lamb et al., 2008 | ‘A major aspect of the recovery experience for elderly individuals who have had a stroke is the considerable physical and psychological work involved in reconstructing their lives.' (p177) |
|  |  | McKevitt et al., 2004Error: Reference source not found | ‘Backe et al (1996) reported that in the first week after stroke, patients’ feelings of unreality and awareness of their changed role might lead to psychological crisis.'(p1500) 'One study reported patients’ accounts of the difficulties they face eating, and the fear and shame this and their changed physical and social appearance created for them. Patients assessed as needing adaptive aids were found to be sometimes reluctant to use such devices because they reinforced the sense of loss associated with stroke impairment. Thus, their unwillingness to use devices was an attempt to avoid stigma rather than noncompliance.' (p1500) |
|  |  | Murray et al., 2003 | ‘The largest domain was social and emotional effects, accounting for 80/203 (39%) of all problems found. Within this domain, mood changes were reported as a problem in over half (15/23) of the studies.' (p138/140) |
|  |  | Peoples et al., 2011 | ‘The individual aspects reflected the stroke survivors’ struggle to cope with and adapt to the impact of stroke, during the process of regaining power and control of their lives.' (p167) |
|  |  | Reed et al., 2012 | ‘All studies were in agreement that the effects of stroke and how they are perceived is highly personalised and dependent on the biography and personal nature of the individual. For example, a stroke could, in part, be accepted by an older person as part of the aging process where ill health might be anticipated, whereas for many people stroke represented a massive disruption in the perceived trajectory of their lives.' (p558) 'Stroke survivors were also shown to compare their current situation with their pre-stroke life. This comparison was perceived as having a negative impact on the stroke survivors who were often described as grieving for their pre-stroke life, and experiencing disappointment when they tried to return to this life.' (p558) |
|  |  | Salter et al., 2008 | ‘Stroke “changed life irrevocably”, and had a significant impact on emotions, personality, abilities, activities, roles and social relationships.' (p597) 'Participants across studies spoke of loss of control, confidence and independence....The disruption in the continuity of perceived self was also noted as a loss of self, or of “pre-stroke me” as well as in a loss of identity associated with the “enforced change in roles”.' (p597-598) 'In addition, helplessness, anger and frustration were described in response to losses of control and independence. “Many felt resigned to a passive role”.' (p598) |
|  | Ambiguity | Lamb et al., 2008 | ‘Initially, there was often uncertainty about the diagnosis and sometimes, delay in seeking treatment as they tried to understand the meaning of the symptoms.' (p177) 'Many experienced a split of the body from the self during this period – their body was separate from themselves, unpredictable and somehow changed.' (p177) |
|  |  | McKevitt et al., 2004 | ‘Shepherd (1994) reported that patients’ misapprehensions about the role of rehabilitation–thought to be convalescent care–was hindering their participation in therapy. Encouraging nurses to educate patients was found to improve their participation.' (p1501) |
|  |  | Salter et al., 2008 | ‘Becker reported that the “future became painfully uncertain” and the body was “the locus of uncertainty”. Early feelings of progress were interrupted by periods of slowed or halted recovery, some wondered if they would ever “get back to normal”. The physical body became unreliable and unpredictable, a source of disappointment.' (p599) |
|  |  | Murray, et al., 2003 | ‘The effects of stroke on self-perception presented as feelings of uncertainty as patients mourned the loss of their identity and independence. (p140) |
|  | New self |  |  |
|  |  | Lamb et al., 2008 | ‘Individuals had to struggle, both psychologically and physically during recovery and to develop strategies to adapt to their new life, often by re-learning and becoming active in their own care.' (p177) |
|  |  | Reed et al., 2012 | ‘Often stroke survivors did not return to pre-existing social activities due to their lower status social role within the groups. The combination of physical and psychological factors led to isolation from the external world. This led to the stroke survivor perceiving themselves as socially redundant.' (p558) |
|  |  | Salter et al., 2008 | ‘The concept of an on-going process of re-interpretation of the self, or of being transformed by stroke, was expressed in the themes and supporting interpretations of the majority of studies. Stroke, and the changes and losses experienced as a result of stroke, challenged the participants’ sense of self and precipitated a sense of discontinuity or estrangement from the person they had been prior to stroke. Participants questioned whether they were the same person at all and struggled to confront perceived changes in an attempt to reconcile their present selves with their pre-stroke selves.' (p597) 'Some participants expressed discomfort with their current selves, in terms of both physical ability and appearance, and became more withdrawn in order to avoid becoming a burden to family, friends and acquaintances.' (p600) |
|  | Environment vulnerability | Reed et al., 2012 | ‘The home was perceived as an important setting for the stroke survivor, a place where they could understand what physical and mental adaptations and adjustments were needed to cope with their stroke. Home also represented a safe place, providing the stroke survivor with an environment that they could be comfortable and confident in. The literature showed that the stroke survivor faced a number of challenges in accessing the world beyond their home, and the external world could be perceived as unsafe. There were three main documented barriers to access: physical, economic and psychological.' (p558) |
| Needs as a result of stroke | Physical | Lamb et al., 2008 | It also involves adapting to changes in physical functioning and to new environments and dealing with the challenges of participating in life activities despite the loss of abilities. A major change is the inability to engage in activities that formed part of former roles, for example, housekeeping activities for a woman who saw these as part of her role as a wife. Coping with physical disabilities involved taking more time to complete daily activities and frequently, the use of physical aids.' (p177) |
|  |  | Peoples et al., 2011 | ‘The participants experienced an overly emphasized focus on rehabilitation of physical needs and a failure to address non-physical needs that could enable the stroke survivors to regain power and control of their lives.' (p168) |
|  |  | Reed et al., 2012 | 'Psychological barriers related to stroke survivors’ negative perceptions of themselves as a disabled person and perceived stigma, which meant difficulties in interacting with the wider public. This varied depending on the social environment the stroke survivor inhabited and other factors, such as age.' (p558) |
|  |  | Salter et al., 2008 | ‘Stroke was seen as something that was always present, mediating experiences, creating effort where there had been none and disrupting the survivors’ sense of self.' (p597) |
|  | Informational | Lamb et al., 2008 | ‘Patients start to seek more information about stroke recovery in the rehabilitation setting….Gaining knowledge helps as a means of controlling their feelings of powerlessness….Information needs remain important after discharge. Elderly patients usually have poor memory and visual problems due to normal ageing.' (p189) |
|  |  | McKevitt et al., 2004 | ‘Needs for information in the longer term have also been reported by many authors, with dissatisfaction expressed about the amount of information provided, the style of delivery, and the timing.' (p1502) 'Respondents were dissatisfied with what had been provided and wanted individualized information related to clinical questions, practical issues, and issues related to services and resources.' (p1502) |
|  |  | Murray et al., 2003 | ‘Lack of written information was an issue reported in just over half of the 23 studies.' (p138) |
|  |  | Peoples et al., 2011 | ‘Information areas of particular importance were: cause of illness, individual progress, evaluation of treatment plan, decisions about discharge and follow-up; “patients explained how information helped them to understand rehabilitation goals and to recognise when they were making progress. This provided reassurance that rehabilitation was working” (Maclean, 2000). Insufficient provision of information preventing them from taking an active part in their rehabilitation: “The lack of information resulted in a feeling of just ‘sitting and waiting for something to happen’” (Röding, 2003).' (p168) |
|  |  | Reed et al., 2012 | ‘Informational support important as gives advice on how to negotiate through the system, emotional support as well.' (p560) 'Need 'external support that could provide information.' (p561) 'Provide practical adaptations and source appropriate levels of support to enable stroke survivors to remain in their own homes; and provide guidance on how to overcome the physical, economic, and psychological barriers in stroke survivors’ external worlds.' (p561) |
|  | Psychological | Lamb et al., 2008 | ‘This work [of recovery] involves drawing on their sense of hope and inner strength or drawing on other attitudes that assist in recovery.' (p177) |
|  |  | McKevitt et al., 2004 | ‘One study investigated patients’ and carers’ problems after stroke to inform the development of a community stroke support service.^99^ Stroke patients’ problems were diverse, complex, and changed over time; initially practical, and, later, psychological.' (p1502) |
|  |  | Peoples et al., 2011 | ‘“The major psychological support valued by the informants included giving them reassurance and a sense of security, offering verbal encouragement, listening to their worries, showing a caring attitude and respecting them as individuals” (Lui, 1999) (32).' (p168) |
|  |  | Reed et al., 2012 | ‘The majority of studies talked about the long-term (multiple year) nature of recovery and issues with coming to terms psychologically with the losses suffered through stroke' (p558) 'The literature contained varying examples of how stroke survivors develop innovative strategies to deal with the effects of stroke in the context of their lives as they strive to maintain control and independence. In some of the studies this desire to maintain control was shown to create a dilemma for the stroke survivor who had difficulty in deciding whether to accept help and adaptations and therefore give up hope of full recovery, or continue with the struggle to live as independently as possible.' (p558) |
|  |  | Salter et al., 2008 | ‘Dowswell et al. suggested that there “appeared to be no fully successful adjustment to stroke” and that the process of adjustment was “bedevilled by constant reference back to life before stroke – not to milestones in recovery”. However, the idea of re-definition and reconciliation in order to create a continuous or coherent sense of self also emerged from the themes and interpretations within the studies. For example, Becker noted that “all respondents searched for anchors of predictability and sought to define and build links between the old self and new imperatives”. Participants in the study by Ellis-Hill et al. were described as “working to get a sort of negotiated settlement – a realignment between body, self and society and create a coherent sense of self”, while Dowswell et al. noted that “a small number of patients had arrived at a sort of truce with themselves”.' (p600) |
|  |  | Murray et al., 2003 | ‘Rehabilitation was considered to be too physically oriented, with deficiencies in social and psychological aspects of recovery. Social services were criticised for the inflexibility of home care, lack of contact with personnel, and lack of advice or guidance on claiming benefits, contributing to financial difficulties. Long delays and broken promises for aids and adaptations were recurring problems.’ (p138) |
|  | Social |  |  |
|  |  | Murray et al., 2003 | ‘Personal relationships were strained because of forced changes in role, overprotective behaviour on the part of the carer, and attempts to maintain a façade of normality.^13,17,26,27^' (p140) |
|  |  | Lamb et al., 2008 | ‘Elderly individuals who have experienced stroke identify the importance of connectedness in their process of recovery.' (p177) 'During the recovery process, connection to others, such as family and friends, spiritual connectedness and relationships with professionals were important for most individuals. The difficulties that might be encountered post-stroke with communication or social activities could lead to an absence of connectedness or a sense of isolation.' (p177) |
|  |  | McKevitt et al., 2004 | ‘A pilot intervention to support stroke survivors in the community, reported that existing problems in the interface between health and social care were such that the intervention was unable to prevent stroke survivors falling between the gaps of service provision.’ (p1502) |
|  |  | Peoples et al., 2011 | ‘The nonphysical needs covered a wide range of areas, e.g. social consequences of stroke, couple counselling, and psychological support.' (p168) 'The opportunity to compare themselves and share feelings and experiences with peers was an important aspect of empowerment: “The patients used the stroke unit in a traditional self-help group manner, to discuss how the stroke came about, how training was going, for mutual encouragement, problem sharing etc.” (Lewinter, 1995).' (p169) |
|  |  | Reed et al., 2012 | ‘Close social support is defined in the studies as family and friends. These relationships are described as providing a “web of support”. This enabled the stroke survivor to adapt to their world, encouraged them not to give up and helped them return to social activities. It also created a safe environment enhancing feelings of belonging and acceptance where stroke survivors could be comfortable with their new selves.' (p558) 'Interaction with the wider social world was perceived as a means of developing a positive trajectory and sense of self post stroke. A number of strategies to enhance social interaction and provide social support for those who want it were documented. These included the use of exercise schemes, a focus on adaption and development of coping skills, external support that could provide information, goal setting to increase independence and confidence, the need for transport and assistive devices to access external events, social comparison with other stroke survivors and, finally, stroke specific groups.' (p561) |
|  |  | Salter et al., 2008 | ‘The importance of relationships, social connections and participation was evident. Relationships supported, comforted and consoled individuals following stroke.' (p599) 'Unfortunately, the majority of participants across studies appeared to experience feelings of increasing social isolation, social withdrawal and/or altered relationships with both families and friends. Participants felt distanced from other people  in that it was “difficult to explain their experiences to others” who found it impossible to “imagine what it must be like to live following a stroke” and found communication with the stroke survivor to be too uncomfortable.' (p600) |
| Experience and impact of services | Recovery plateau | McKevitt et al., 2004Error: Reference source not found | ‘Nevertheless the process of adjustment after stroke has been described as difficult and slow, with plateaus in recovery presenting survivors with unexpected obstacles.' (p501) ‘Attempts to draw on patients’ accounts to delineate phases of the stroke trajectory have been made, with the argument that services as currently configured do not adequately reflect survivors’ experience of trying to adjust to the effects of stroke.' (p501) |
|  |  | Salter et al., 2008 | ‘Initial optimism regarding progress toward these goals tended to diminish over time, particularly as recovery slowed or stalled.' (p600) |
|  | Respect and value | Lamb et al., 2008 | ‘Although for the most part relationships with professionals are positive during the recovery process, some elderly individuals perceived a lack of respect. A respectful approach, encouragement and reassurance from health professionals were important to those who had experienced a stroke.' (p177) |
|  |  | McKevitt et al., 2004 | ‘The [specialist nurse support] intervention was valued for the “less tangible” aspects of nursing care it provided: concern, attention, empathy, and interest, combined with emotional and practical support.' (p1502) |
|  |  | Peoples et al., 2011 | ‘Attitudes from the staff greatly affected the stroke survivors’ ability to maintain their individuality and dignity. Issues of importance were: attentiveness, respect and support, being properly addressed, and having personal choices valued and respected.' (p168) 'Disrespectful interactions led to feelings of subordination and disempowerment.' (p168) |
|  |  | Salter et al., 2008 | ‘This separation from others seemed to promote a climate in which the stroke survivors felt as though they were misunderstood or even treated rudely or dismissively because of their disabilities, both visible and invisible.' (p600) |
|  | Communication | Lamb et al., 2008 | ‘During the period of recovery, there was often uncertainty about the extent of recovery and individuals tended to measure their progress in terms of their prestrike life, although they perceived that professionals measured progress differently.' (p177) |
|  |  | Lui et al., 2005 | ‘These studies shed light on unrealistic goal setting and help explain discrepancies between caregiver and patient perceptions of outcome; for example, in some cases where caregivers could identify that some goals were met, patients may have focused on what they could not yet do rather than what they had achieved.' (p2519) |
|  |  | McKevitt et al., 2004 | ‘Contrasting perceptions of rehabilitation were described: for patients, rehabilitation suggested ability to recover if they worked hard enough, resulting in feeling let down when recovery did not occur.' (p1501)  'Mismatches between professional and patient goals as well as defects in the organization of existing services leading to setbacks in patient recovery.' (p1501) 'While professionals measure recovery in terms of regaining function, for patients this can mean return to prestroke life. It has also been suggested that recovery is defined by patients in the relation to their own social context and in terms of achieving their own goals. Thus, current methods of assessing progress after stroke have been criticized for failing to take into account patients’ ideas about what recovery means to them.' (p1501) |
|  |  | Murray et al., 2003 | ‘Deficiencies in communication, mostly relevant written information, were commonly reported by patients and carers.' (p138) 'With regard to primary care, patients and carers were unhappy about the quantity and quality of general practitioner (GP) contacts. Two studies reported on an expectation of home monitoring visits, which rarely occurred. Other studies found high rates of GP contact, but patients still felt that they lacked medical supervision. Further concerns related to a perceived lack of interest and stroke knowledge.' (p138) |
|  |  | Peoples et al., 2011 | ‘The participant’s experiences of collaboration with staff were diverse, covering a collaboration continuum with shared decision-making and paternalism as two contrasting end points.' (p168) '“Patients mostly value paternalism regarding treatment decisions. . ..However, they do not appreciate paternalism with regard to other decisions, for example, regarding toilet times and leisure activities” (Proot, 2000). Insufficient collaboration resulted in a feeling of being disconnected and passive: “The understanding of the rehabilitation process was very vague and the informants felt they were ‘walking alongside’ the process” (Röding, 2003).' (p168) |
|  | Proactivity | Lamb et al., 2008 | ‘Spiritual connection seemed to provide a sense of confidence about the future.' (p177) 'Individuals had to struggle, both psychologically and physically during recovery and to develop strategies to adapt to their new life, often by re-learning and becoming active in their own care.' (p177) |
|  |  | Lui et al., 2005 | ‘The results suggest that both the patients and caregivers appreciated nurses as proactive, friendly, and encouraging.' (p2519) 'For the goal-setting process, all of the nurses stressed the use of a collaborative approach with the patients and their caregivers.' (p2519) |
|  |  | McKevitt et al., 2004 | ‘Home-based rehabilitation encouraged a partnership between the patient and therapist, and provided the opportunity to discuss rehabilitation issues and give advice and teaching in the family setting. Early discharge had a positive impact on rehabilitation, encouraging patients to practice activities on their own and devise their own solutions to problems. The therapy was more relevant to the patients’ needs in their own environment.' (p1501) 'It has been reported that survivors regard following professional advice, making their own care decisions, and staying positive and motivated as important. Survivors have also been found to develop their own strategies to combat disabilities, including tackling tasks more slowly and initiating learning and exercise, developing strategies to maintain or re-establish a sense of continuity after the disruptive life event that stroke represents, using strategies to foster hope during the process of adjusting to life after stroke, and drawing on spiritual practices.' (p1501) |
|  |  | Murray et al., 2003 | ‘Therapy-related problems included: lack of longer-term contact and social guidance; inappropriate goal setting; and limited access to, or unawareness of, services.' (p138) |
|  |  | Peoples et al., 2011 | ‘Participants reported a need for active participation in their rehabilitation. This was achieved through awareness of their situation and by being engaged in independent activities where they could regain a feeling of control of their situation.' (p168) 'However, not all participants were ready to assume power and responsibility and the demands placed upon them was perceived as frustrating.' (p169) |
|  |  | Reed et al., 2012 | ‘The key factors to be considered in supporting stroke survivors and helping them maintain an active and positive presence in their unique social world are to: 1. identify personally relevant goals of stroke survivors and their carers, to enable personal control and independence; 2. provide practical adaptations and source appropriate levels of support to enable stroke survivors to remain in their own homes; 3. provide guidance on how to overcome the physical, economic, and psychological barriers in stroke survivors’ external worlds; and 4. enhance internal confidence by supporting positive social interaction.' (p561) |
|  |  | Salter et al., 2008 | ‘From the backdrop of change and transformation, the ideas of resiliency and adaptation emerged. As time passed from the stroke event, some informants began to focus on more positive aspects of their lives over which they had regained a sense of control. For example, some participants “spoke of experiences in which they were in control…these included mastering new skills, adapting old skills and changing their environment”. For some, this process of re-focusing and adaptation was associated with feelings of hope for recovery.' (p600) |
